# Inhibitory Effect of *Nelumbo nucifera* Leaf Extract on 2-Acetylaminofluorene-induced Hepatocarcinogenesis Through Enhancing Antioxidative Potential and Alleviating Inflammation in Rats

**DOI:** 10.3390/antiox8090329

**Published:** 2019-08-22

**Authors:** Mon-Yuan Yang, Tung-Wei Hung, Chau-Jong Wang, Tsui-Hwa Tseng

**Affiliations:** 1 Institute of Biochemistry, Microbiology and Immunology, Chung Shan Medical University, Taichung 40201, Taiwan; 2 Division of Nephrology, Department of Medicine, Chung Shan Medical University Hospital, Taichung 40201, Taiwan; 3 School of Medicine, Chung Shan Medical University, Taichung 402, Taiwan; 4 Department of Health Diet and Industry Management, Chung Shan Medical University, Taichung 40201, Taiwan; 5 Department of Medical Research, Chung Shan Medical University Hospital, Taichung 40201, Taiwan; 6 Department of Medical Applied Chemistry, Chung Shan Medical University, Taichung 40201, Taiwan; 7 Department of Medical Education, Chung Shan Medical University Hospital, Taichung 40201, Taiwan

**Keywords:** 2-acetylaminofluorene, antioxidant enzymes, hepatocellular carcinoma, inflammation, *Nelumbo nucifera* leaf extract, oxidative stress

## Abstract

Leaf extract of *Nelumbo nucifera* (NLE) has been demonstrated to possess anti-atherosclerosis, improve alcohol-induced steatohepatitis, prevent high-fat diet-induced obesity, and inhibit the proliferation and metastasis of human breast cancer cells. This study determines the chemopreventive role of NLE against 2-acetylaminofluorene (AAF)-induced hepatocellular carcinoma (HCC) in rats. AAF was used to induce hepatocarcinogenesis in rats through genetic and nongenetic effects. After administration for 12 weeks, NLE (0.5–2%) supplementation orally inhibited AAF (0.03%)-induced hepatic fibrosis which appears during the development of premalignant lesions in rats. After the 6-month experiment, NLE supplementation resulted in decreasing AAF-induced serum parameters of hepatic injury, including the level of triglycerides, total cholesterol, alpha-fetoprotein (AFP), and inflammatory mediator interleukin (IL)-6 and tumor necrosis factor (TNF)-α as well as the activities of alanine aminotransferase (ALT), aspartate aminotransferase (AST), and gamma-glutamyl transferase (γGT). NLE supplementation also reduced AAF-induced lipid peroxidation and 8-hydroxy-2′-deoxyguanosine (8-OHdG) formation in the rat liver. Hepatic histopathological investigation revealed that NLE supplementation attenuated the AAF-induced HCC and glutathione S-transferase-Pi (GST-Pi) expression. Furthermore, NLE supplementation increased the expression of transcription factor, nuclear factor erythroid 2-related factor 2 (Nrf2) and its downstream targets, including catalase, glutathion peroxidase (GPx), and superoxide dismutase 1 (SOD-1) in the rat liver. Our findings indicate that NLE supplementation inhibited AAF-induced hepatocarcinogenesis by enhancing antioxidative potential and alleviating inflammation in rats.

## 1. Introduction

Hepatocellular carcinoma (HCC) is a common malignant tumor and has a high mortality rate worldwide. Because most HCC patients are frequently diagnosed at an advanced stage, the overall 5-year survival rate of HCC is estimated to be below 20% [1]. In addition, the recurrence rate can be as high as 50%. The development of HCC is a complex process involving the accumulation of genetic and epigenetic alteration, which passes through states of initiation, promotion, and progression, with both viral and chemical carcinogens involved in the multistate process. The onset of HCC is usually late in patients with chronic liver diseases such as hepatitis B and C infections and dietary carcinogen exposure. Accordingly, hepatic fibrosis occurs when liver is repeatedly and continuously injured. Damage to hepatocytes triggers the release of reactive oxygen species (ROS) and inflammatory mediators, which would stimulate carcinogenic process [2]. Untreated fibrosis may progress to liver cirrhosis, leading to organ failure and hepatoma [3,4]. Therefore, approaches on antioxidant, anti-inflammation, and anti-fibrosis are considered the effective strategy for lowering the morbidity and mortality of HCC.

*Nelumbo nucifera* Gaertn (*Nelumbonaceae*), commonly referred to as lotus, is a widely distributed crop in central and southern Taiwan. In Southeastern Asia, lotus seed and rhizomes are usually considered popular dietary staples, and lotus leaf is also used as a common food item in Taiwan. Additionally, all parts of *N. nucifera*, including the leaves, flowers, stamens, embryos, and rhizomes, have been used as medicinal herbs in Eastern Asia and exhibit pharmacological properties such as antipyretic, antidiabetic, antioxidative and antiobesity effects [5,6,7,8,9,10]. In addition, *Nelumbo nucifera* leaf extract (NLE) was demonstrated to have remarkable effectiveness in reducing the development of atherosclerosis [10], preventing high-fat diet-induced obesity and body fat accumulation [11], improving alcohol-induced steatohepatitis [12], and inhibiting the proliferation and metastasis of human breast cancer cells [13,14]. Moreover, our study found that NLE alleviated high-fat diet-induced hepatic injuries in hamsters [15]. Our previous study demonstrated that NLE contains phenolic compounds including phenolic acid and flavonoids [12,13]. In related studies, gallic acid induces HepG2 cell apoptosis [16] and protects against diethylnitrosamine-induced rat hepatocellular carcinoma [17]. Catechin prevents the development of hepatic neoplasma by modulating several signal transduction and metabolic pathways [18]. Rutin suppresses epidermal growth factor receptor (EGFR) kinase activity [19] and inhibits N-nitrosodiethylamine-induced HCC in Wistar rats [20]. Isoquercitrin inhibits the growth of HepG2 cells and xenograft tumor growth in nude mice [21]. Miquelianin promots doxorubicin-induced apoptosis under endoplasmic reticulum (ER) stress in HCC cells [22]. Astragalin reduces the proliferation of HCC cells and inhibits the growth of xenograft tumors in vivo [23]. These reports imply that NLE may exert antihepatoma potential. However, the protective effects of NLE on oxidative stress-triggering hepatocarcinogenesis remain unclear. Therefore, we investigated the effects of NLE on hepatocarcinogenesis by 2-acetylaminofluorene AAF, which can induce liver cancer through genotoxic and nongenotoxic process associated with promoting oxidative stress in rats [24,25].

## 2. Materials and Methods 

### 2.1. Chemicals and Reagents

The leaves of *N. nucifera* Gaertn. were purchased from Paiho Farmers’ Association Organization in Tainan County, Taiwan. Formic acid (reagent grade, 96%) was purchased from Tedia (Fairfield, OH, USA). Acetonitrile and methanol (high-performance liquid chromatography-grade, Darmstadt, Germany). The liquid chromatography grade polyphenol standards including isoquercitrin, peltatoside, and miquelianin were obtained from Extrasynthese (Genay, France). AAF, bovine serum albumin, dithiothreitol, ethylenediaminetetraacetic acid, formaldehyde, N-(2-hydroxyethyl) piperazine-N’-ethanesulfonic acid, phenylmethylsulfonyl fluoride, and sodium phosphate were purchased from Sigma (St. Louis, MO, USA). Antibodies against catalase, glutathion peroxidase (GPx), superoxide dismutase 1 (SOD-1), and nuclear factor erythroid 2-related factor 2 (Nrf2) were purchased from Cell Signaling Technology (Beverly, MA, USA), and β-actin as internal control was purchased from Santa Cruz Biotechnology (Santa Cruz, CA, USA). Peroxidase-conjugated antibodies against rabbit IgG or mouse IgG were purchased from Sigma.

### 2.2. Preparation of NLE and HPLC Analysis

NLE was prepared using a method similar to that in our previous paper and analyzed using HPLC [12,13]. Briefly, dried *N. nucifera* leave (20 g) was resuspended in 100 mL of distilled water at 4 °C for 12–16 h. The supernatant was filtered and concentrated under reduced pressure. Finally, the concentrated solution was lyophilized as NLE. HPLC analysis were using a Waters system which consisted of a HPLC controller and pump (Waters 600), a detector (2998 PDA) and an in-line degasser AF. A systemic procedure for analyzing the polyphenols contained a Mightysil RP-18 GP 250 column (Kanto, Tokyo, Japan), mobile phase solvent A (formic acid in water, pH = 2.5), solution B (acetonitrile) and solution C (methanol). HPLC method was flowing with gradient program: 0 min, 100% A; 5 min, 90% A, 0% B; 15 min, 85% A, 3% B; 20 min, 85% A, 5% B; 30 min, 85% A, 8% B; 50 min, 85% A, 15% B; 65 min, 75% A, 25% B; 70 min, 70% A, 30% B; 80 min, 50% A, 50% B; 85 min, 100% B; 100 min, 100% B. Flow rate was 1.0 mL/min and injection volume was 20 μL. The UV detection range was on 210–400 nm.

### 2.3. Animals Maintenance and Treatment 

Male Wistar rats, aged 4–5 weeks and weighing 140–160 g, were purchased from the National Laboratory Animal Center (Taipei, Taiwan), kept at a constant temperature between 22 and 24 °C with a 12 h light/12 h dark cycle. All protocols were approved by the Institutional Animal Care and Use Committee of Chung Shan Medical University (IACUC, CSMU, approval no. 1070). Rats were fed with Laboratory Rodent Diet 5001 (PMI Nutrition International) containing 23.0% crude protein, 4.5% crude fat, 6.0% crude fiber, and 8.0% ash, as described in the manufacturer’s instructions. Before the randomly grouped by body weight, rats were monitored for adaptation to the environment at the first week. Diets and drinking water were provided ad libitum to rats all through the experiment. We provided 20 g per rat for the first week and changed the amount of diet in accordance with the change of the bodyweight weekly. The weight of intake diet of each cage was measured at least once a week and the average of diet consumption of each group per rat was estimated monthly as one of monitors of physiological response. For AAF-induced HCC [26], the rats were fed with 0.03% AAF in daily diet and divided into four groups. According to IACUC guidelines, there were two rats in a separated cage which were fed a unique diet for six months and detected the body weight monthly. Rats fed with AAF-diet to induced HCC were divided into five groups as follows: (1) normal diet as control group (2) AAF, the normal diet containing 0.03% AAF (*w*/*w*); (3) AAF + 0.5% NLE, the AAF-diet containing 0.5% NLE (*w*/*w*); (4) AAF + 1% NLE, the AAF-diet containing 1% NLE (*w*/*w*); and (5) AAF + 2% NLE, the AAF-diet containing 2% NLE (*w*/*w*). Since liver fibrosis is strongly associated with HCC, with 90% of HCC cases arising in cirrhotic livers [2], we also have five groups as control, AAF, AAF + 0.5% NLE, AAF + 1% NLE and AAF + 2% NLE group, which were examined the liver fibrosis level of rats after three months of application of different diets. Rats from each group were sacrificed for the pathological determination of liver fibrosis. After six months of application of different diets, blood and whole liver samples were collected from rats (n = 10) that had been fasted for 12–14 h and then sacrificed. The whole livers were pictured, detected the body weight, and extracted the proteins.

### 2.4. Histopathological Examination for Malignant Hepatoma

The livers were collected and fixed in 10% buffered neutral formalin, then embedded in paraffin. The section was cut at a thickness of 3–5 μm and stained with hematoxylin and eosin (H&E) and with Masson’s trichrome for the detection of collagen fibers. The histopathological changes such as cellular lipid vesicles and cell morphology were examined through light microscopy. 

### 2.5. Immunohistochemical Evaluation

Liver sections were deparaffinized in xylene, rehydrated in a graded alcohol series, and blocked with 3% H_2_O_2_ or 5% bovine serum albumin in methanol for 10–30 min. Tissue sections were washed with phosphate buffer solution and then immunostained with primary antibodies for glutathione S-transferase-Pi (GST-Pi) and 8-hydroxy-2′-deoxyguanosine (8-OHdG). Sections were then incubated with horseradish peroxidase-conjugated secondary antibodies, washed, covered with 3,3′-Diaminobenzidine, and counterstained with hematoxylin.

### 2.6. Determination of Serum Biomarkers for Liver Fibrosis and Hepatocarcinogenesis

Blood serum was obtained by centrifuging at 1000 × g for 10 min. Total triglycerides (TR213, Randox Laboratories, Antrim, UK), total cholesterol (CH201, Randox Laboratories, Antrim, UK), aspartate aminotransferase (AST) (AS521, Randox Laboratories, Antrim, UK), alanine aminotransferase (ALT) (AL520, Randox Laboratories, Antrim, UK), gamma-glutamyl transferase (γGT) (GT523, Randox Laboratories, Antrim, UK), and alpha-fetoprotein (AFP) (AB360, Randox Laboratories, Antrim, UK), were determined using by enzymatic colorimetric methods using commercial kits (Randox Laboratories, Antrim, UK), respectively [27]. 

### 2.7. Measurement of Lipid Peroxidation and Antioxidant Enzymes

Lipid peroxidation was determined by measuring homogenate malondialdehyde (MDA) concentration which was reacted with thiobarbituric acid to form thiobarbituric acid reactive substances (TBARS) [28]. The 532 nm absorbance of the supernatant was measured. The results were presented as TBARS level mmol/mg protein. The rat liver frozen tissue was homogenized in ice-cold phosphate buffer (pH = 7.4) and centrifuged at 3000 rpm for 10 min. The concentration of protein (mg/mL) from the liver was determined by the biuret method. A standard curve was established with serial dilutions of the bovine albumin solution. Spectrophotometric absorbance was determined in the supernatant as 595 nm [29]. GPx converts reduced glutathione (GSH) into oxidized form using H_2_O_2_. The amount of GSH utilized was measured in the assay mixture before and after the enzyme activity. GSH-Px activity (mM GSH oxidized/min/mg protein) was determined spectrophotometrically according to the method of Lawrence and Burk [30]. The SOD activity was conducted using a modified method described previously [31]. SOD activty were measured by pyrogallol autoxidation assay. SOD activity (U/mg protein) was calculated based on the inhibition of 0.2 mM pyrogallol autooxidation (U = 50% inhibition of activity). Catalase activity (mM H_2_O_2_/min/mg protein) was measured with 0.03 M H_2_O_2_ at 240 nm based on Aebi [32] with some modifications.

### 2.8. Western Blot Analysis

Total protein of livers derived from AAF-induced rats were extracted with protein extraction solution (Cat. no. 17081, iNtRON Biotechnology, Seongnam, Korea) as manufactory’s protocals. Sixty μg proteins were separated with sodium dodecyl sulfate polyacrylamide gel electrophoresis (SDS-PAGE) and electrotransferred into nitrocellulose membranes (Millipore, Billerica, MA, USA). The membranes were blocked with phosphate buffer saline containing 5% non-fat milk, probed with specific first antibodies, and then incubated with horseradish peroxidase conjugated second antibody (GE Healthcare, Buckinghamshire, UK). The densitometry of protein band was evaluated with Fujifilm Multi Gauge version 2.2 software.

### 2.9. Assay of Serum IL-6 and TNF-α

Serum proinflammatory cytokine levels were determined by high sensitivity IL-6 and TNF-α commercial kits. These measurements were based on a solid-phase sandwich enzyme-linked immunoassay with recombinant human IL-6 (ELR-IL6, RayBiotech, Atlanta, GA, USA), TNF-α (ELR-TNFα, RayBiotech, GA, USA).

### 2.10. Statistical Analysis 

The experimental results are expressed as means with their standard deviations [SDs]). The data for the experimental groups were firstly tested for normality of distribution with the Shapiro-Wilk test. The animal results were statistically analyzed by one-way analysis of variance (ANOVA) and significantly different at *p* < 0.05 according to Duncan’s multiple range test (Sigma-Plot 12.0, Jandel Scientific, San Rafael, CA, USA).

## 3. Results

### 3.1. Identification of NLE

The presence of polyphenol as the major phytochemicals of *N. nucifera* was confirmed by high-performance liquid chromatography (HPLC) analysis as our previous paper [12,13]. Six phenolic compounds were used as the standard, and the quantitative results revealed that NLE contained gallic acid (6.11 ± 0.15 μg/mg NLE), catechin (7.42 ± 0.69 μg/mg NLE), peltatoside (3.55 ± 0.04 μg/mg NLE), rutin (4.65 ± 0.14 μg/mg NLE), isoquercitrin (2.83 ± 0.10 μg/mg NLE), miquelianin (8.81 ± 0.18 μg/mg NLE), and astragalin (0.75 ± 0.05 μg/mg NLE) through comparison of HPLC retention times with corresponding standards.

### 3.2. Effect of NLE Supplementation on AAF-Induced Fibrosis 

**Figure 1:**
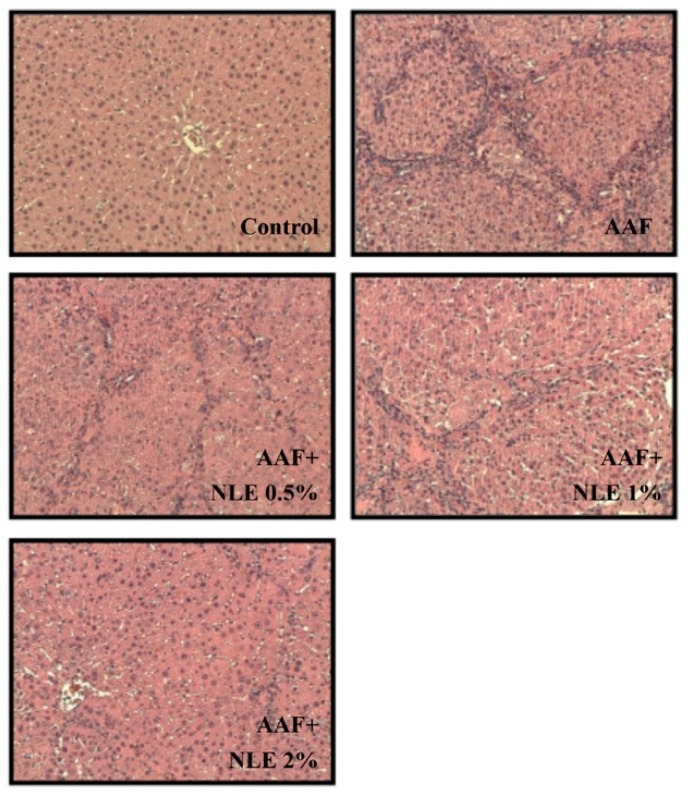
Effect of *Nelumbo nucifera* (NLE) supplementation on 2-acetylaminofluorene (AAF)-induced liver fibrosis in rats. Rats were fed a normal diet (as the control group), a normal diet containing AAF (as the AAF group), a normal diet containing AAF with 0.5% NLE (as the AAF + NLE 0.5% group), a normal diet containing AAF with 1% NLE (as the AAF + NLE 1% group), and a normal diet containing AAF with 2% NLE (as the AAF + NLE 2% group) for 12 weeks. Collagen fibers were stained by Masson’s trichrome (blue).

Because liver fibrosis has been reported to be strongly associated with HCC [2], we examined the liver sections of rats after three months of treatment with AAF through Masson’s trichrome staining. The control group has no fibrosis. By contrast, numerous blue collagen fibers were observed in the AAF-treated rats (Figure 1). The groups with NLE supplementation exhibited a reduction in fiber extension in a dose-dependent manner.

### 3.3. Body and Liver Weight and Exterior Examination 

**Figure 2:**
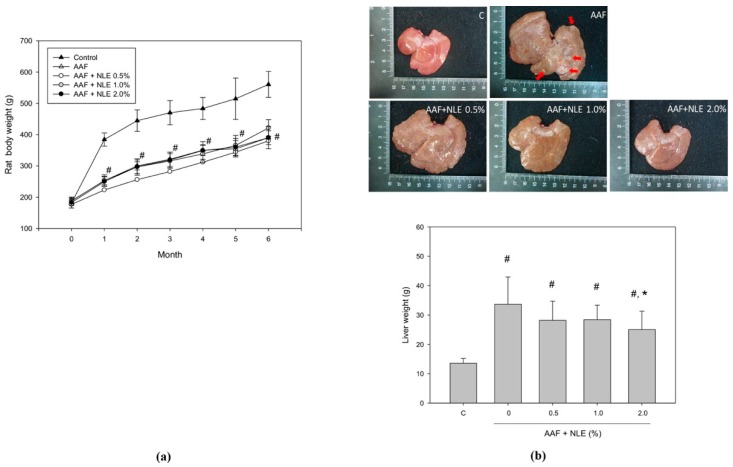
Effect of NLE supplementation on body weight and liver weight in AAF-induced rats. Wistar rats were fed a normal diet (as the C group), a normal diet containing AAF (as the AAF group), normal diet containing AAF (0.03%) with 0.5% NLE (as the AAF + NLE 0.5% group), a normal diet containing AAF with 1% NLE (as the AAF + NLE 1% group), and a normal diet containing AAF with 2% NLE (as the AAF + NLE 2% group) for six months. (**a**) Changes in the body weight of rats in each month. (**b**) After 6 months, the rats were sacrificed and their liver weights were determined. Data are shown as means ± SDs (n = 10); # *p* < 0.05, compared with the control group and * *p* < 0.05, compared with the AAF group.

The variation in body weight during the 6-month experiment is shown in Figure 2a. AAF treatment significantly suppressed the increase in body weight compared to the control; this finding indicated that the growth of AAF-treated rats was prominently suppressed. Although NLE supplementation did not alleviate AAF-induced body weight loss, it reduced AAF-induced enlargement of the rat liver (Figure 2b). Moreover, AAF treatment increased the liver weight by 2.7-fold in the AAF plus NLE supplementation group compared to the control group. A 2% NLE supplementation significantly reduced the liver weight in the AAF plus NLE supplementation group compared to the AAF-treatment-only group. 

### 3.4. Blood Biochemical Study for Hepatic Injury 

**Figure 3:**
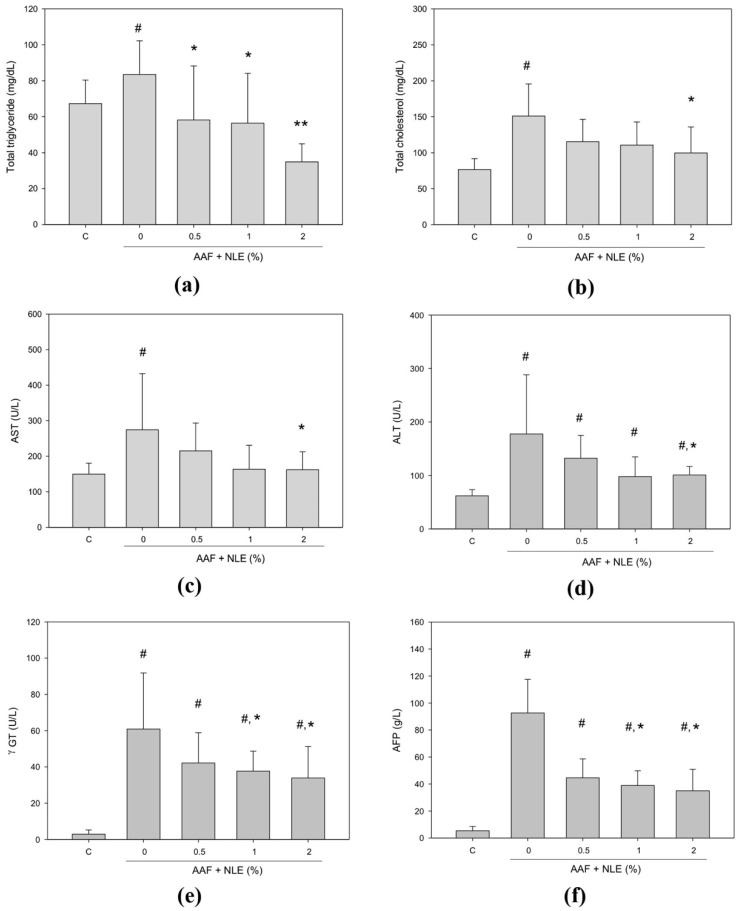
Effect of NLE supplementation on the serum biomarkers of hepatic injury and hepatocellular carcinoma in AAF-induced rats. The biomarkers quantitated were as follows: (**a**) total triglyceride level, (**b**) total cholesterol level, (**c**) aspartate aminotransferase (AST) activity, (**d**) alanine aminotransferase (ALT) activity, (**e**) gamma-glutamyl transferase (γGT) activity, and (**f**) alpha-fetoprotein (AFP) level in plasma from AAF-induced rats. C, a normal diet; AAF, a normal diet containing AAF; AAF + NLE 0.5%, a normal diet containing AAF with 0.5% NLE; AAF + NLE 1%, a normal diet containing AAF with 1% NLE; and AAF + NLE 2%, a normal diet containing AAF with 2% NLE. Data are shown as means ± SDs; Results were statistically analyzed with ANOVA and significantly different was set as *p* < 0.05 according to Duncan’s multiple range test. # *p* < 0.05, compared with the control group and * *p* < 0.05, and ** *p* < 0.01, compared with the AAF group.

After the 6-month experiment, five groups of rats were sacrificed, and the changes in serum makers for hepatic injury induced by AAF were examined. As shown in Figure 3a–f, AAF treatment significantly increased the levels of total triglycerides, total cholesterol, AFP and the activities of AST, ALT, γ-GT, in the AAF-treated rats compared to the control rats. Elevated levels of serum total triglyceride and cholesterol are associated with liver function disorder; elevated activities of both AST and ALT indicate liver injury; and elevated activity of γ-GT and the level of AFP are associated with increased liver cancer risk. Notably, 2% NLE supplementation significantly reduced the levels of serum total triglyceride, total cholesterol, AFP and the activities of AST, ALT, γ-GT induced by AAF. Moreover, 1% NLE supplementation significantly reduced the levels of total triglycerides, AFP, and the activity of γ-GT, induced by AAF (Figure 3). Interestingly, although NLE supplementation decreased the activity of γ-GT, and the level of AFP compared with AAF-fed group, but the activity of γ-GT, and the level of AFP still higher than control group.

### 3.5. NLE Supplementation Inhibits AAF-Induced Inflammatory Mediators 

**Figure 4:**
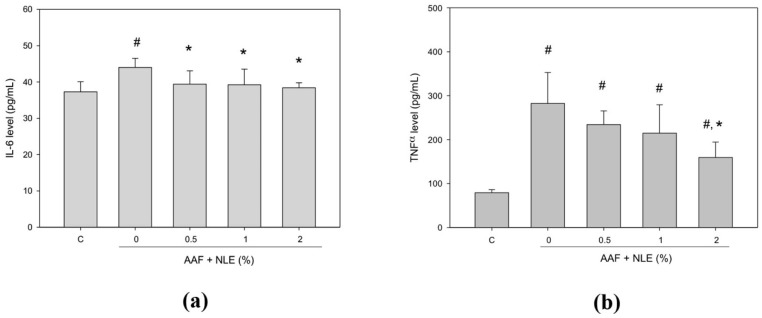
Effect of NLE supplementation on the inflammatory mediators induced by AAF in rats. The inflammatory factors interleukin (IL-6) (**a**) and tumor necrosis factor (TNF-α) (**b**) levels were quantitated in the plasma from AAF-induced rats. Rats were fed a normal diet (as the C group), a normal diet containing AAF (as the AAF group), a normal diet containing AAF with 0.5% NLE (as the AAF + NLE 0.5% group), a normal diet containing AAF with 1% NLE (as the AAF + NLE 1% group), and a normal diet containing AAF with 2% NLE (as the AAF + NLE 2% group). Data are shown as means ± SDs; Results were statistically analyzed with ANOVA and significantly different was set as *p* < 0.05 according to Duncan’s multiple range test. # *p* < 0.05, compared with the control group, * *p* < 0.05 compared with the AAF group.

Serum biochemistry data (Figure 3) demonstrated that AAF induced hepatic damage or inflammation. Thereafter, we evaluated the levels of IL-6 and TNF-α induced by AAF in the rat livers. As shown in Figure 4, AAF significantly increased the levels of IL-6 and TNF-α in AAF-treated rats compared with the control, whereas the 2% NLE supplementation group exhibited a reduction in cytokine levels induced by AAF. Moreover, although NLE supplementation decreased the level of TNF-α compared with AAF group, but the level of TNF-α still higher than control group.

### 3.6. Effect of NLE on AAF-Induced Hepatocarcinogenesis

**Figure 5:**
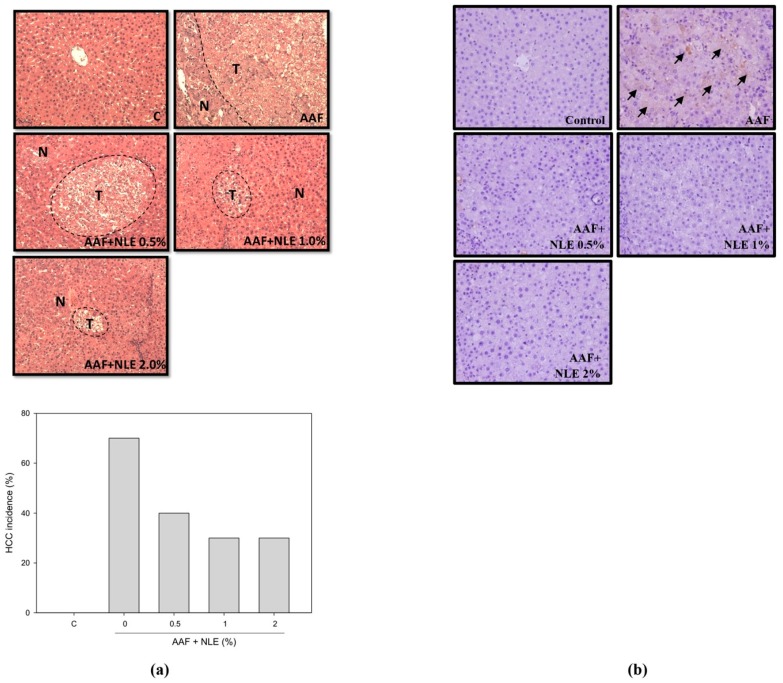
Effect of NLE supplementation on AAF-induced HCC. (**a**) The sections were stained with H&E and examined under a microscope at 100×. N, normal hepatocyte; T, tumor. Livers from rats fed a normal diet (as the C group), a normal diet containing AAF (as the AAF group), a normal diet containing AAF with 0.5% NLE (as the AAF + NLE 0.5% group), a normal diet containing AAF with 1% NLE (as the AAF + NLE 1% group), and a normal diet containing AAF with 2% NLE (as the AAF + NLE 2% group) were fixed, embedded, and sectioned. The incidence of hepatocellular carcinoma with or without NLE supplementation in AAF-induced rats was calculated. (**b**) Effect of NLE supplementation on the expression of glutathione S-transferase-Pi (GST-Pi) induced by AAF in the liver of rat (arrow head). Livers from Wistar rats were fixed, embedded, and sectioned. The sections were stained with anti-GST-Pi and examined under a microscope at 200×.

Chronic inflammation can alter cytokine expression within the injured liver, leading to an excessive increase in reactive oxygen species (ROS), which contributes to HCC initiation and progression. We evaluated whether AAF may induce hepatocarcinogenesis after 6 months of treatment through a liver pathology examination. Compared with the control rat liver, the tissue section of the AAF-treatment-only rat showed many abnormal hepatocytes with a high nucleoplasm/cytoplasm ratio, a typical morphology of HCC (Figure 5a); 70% of the AAF-treated rats presented hepatic carcinogenesis and exhibited severely abnormal hepatocyte morphology. Compared with the AAF-treatment-only rats, the hepatic carcinogenesis incidence in rats with 0.5%, 1%, and 2% NLE supplementation decreased up to 40%, 30%, and 30%, respectively. Increased expression of hepatic GST-Pi is a sensitive marker for oxidative stress, which is causally related to carcinogenesis in the liver [33]. In the present study, numerous GST-Pi-positive foci were found in the liver of AAF-treatment-only rats (Figure 5b), whereas no GST-Pi-positive foci were found in either the control group or the AAF plus 2% NLE supplementation group.

### 3.7. NLE Supplementation Enhances Antioxidant Potential

**Figure 6:**
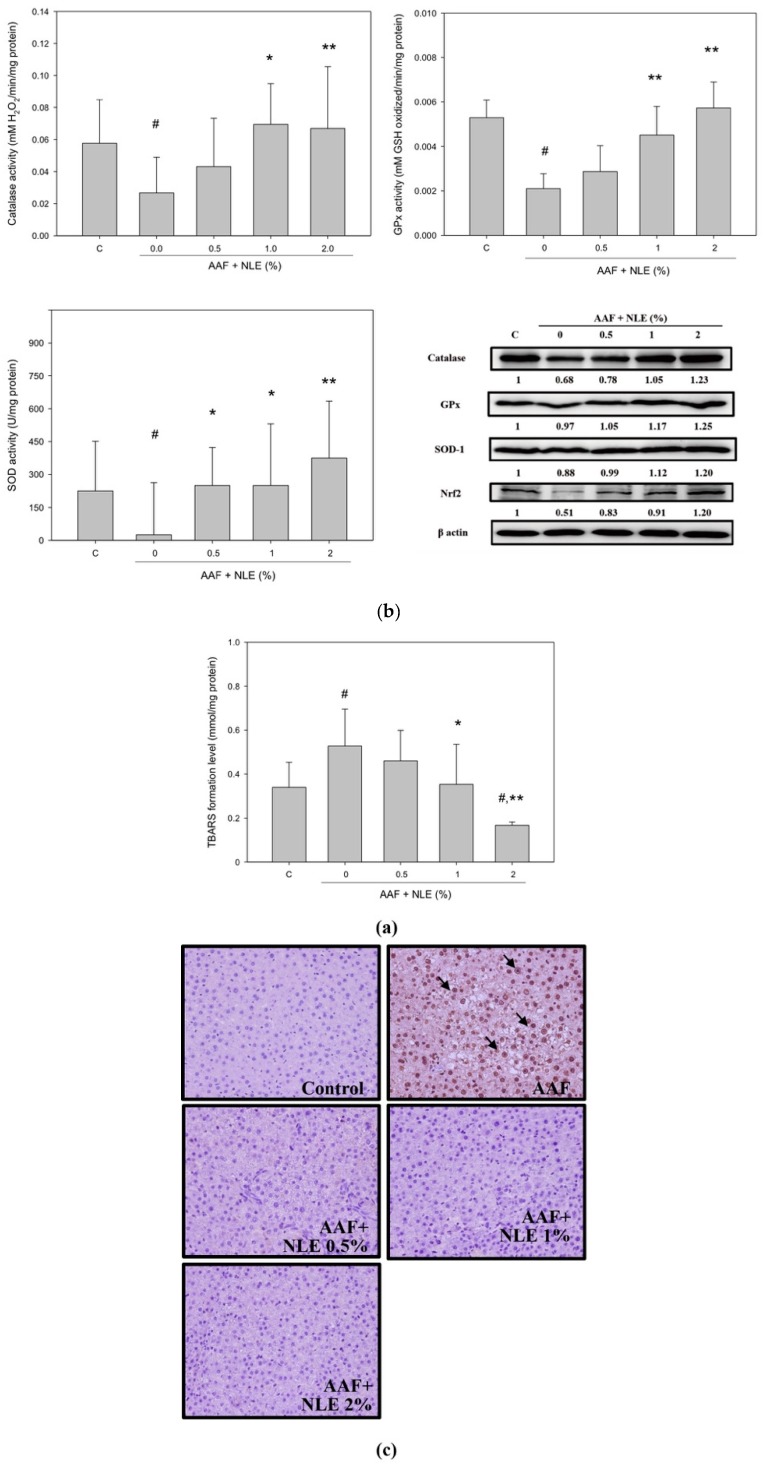
Effect of NLE supplementation on oxidative stress induced by AAF in the rat liver. (**a**) Levels of thiobarbituric acid reactive substances (TBARS) formation in the liver were quantitated through spectrophotometric analysis. (**b**) glutathion peroxidase (GPx), superoxide dismutase 1 (SOD-1), and catalase enzymatic activity in the liver was quantitated through spectrophotometric analysis. Data are presented as mean ± SD from three independent experiments. Results were statistically analyzed with ANOVA and significantly different was set as *p* < 0.05 according to Duncan’s multiple range test. # *p* < 0.05, compared with the control group, * *p* < 0.05 and ** *p* < 0.01, compared with the AAF group. Liver tissue extracts were subjected to Western blotting to analyze catalase, GPx, SOD-1, and nuclear factor erythroid 2-related factor 2 (Nrf2) expression. The levels of these proteins were subsequently quantitated through densitometric analysis with the control being 100%. (**c**) Livers from Wistar rats were fixed, embedded, and sectioned. Sections were stained with anti-8-hydroxy-2′-deoxyguanosine (8-OHdG) and examined under a microscope at 200×.

Lipid peroxidation is a critical marker for oxidative stress and is coupled with various diseases including cancer. As shown in Figure 6a, AAF treatment significantly increased the TBARS formation of liver tissue compared with the control group, whereas 1% and 2% NLE supplementation significantly blocked AAF-induced TBARS. Furthermore, AAF treatment reduced the activities and protein levels of catalase, GPx, and SOD-1 compared to the control group. NLE supplementation (1% and 2%) significantly restored the activities and protein levels of catalase, GPx, and SOD-1 in the AAF-treatment group (Figure 6b). Nrf2 is a transcription factor associated with the expression of antioxidant enzymes; therefore, the effect of NLE supplementation on the expression of Nrf2 was evaluated. As shown in Figure 6b, AAF treatment inhibited Nrf2 expression in the rat liver. The quantitated level of Nrf2 revealed that AAF treatment diminished the expression of Nrf2 to 51% compared with the control. NLE supplementation at concentrations of 0.5% and 1% increased the expression level of Nrf2 compared with AAF treatment alone, and 2% NLE supplementation was improved the 20% expression level of Nrf2 than that in the control (Figure 6b). Because 8-OHdG has been used as a pivotal marker for endogenous oxidative stress and carcinogenesis, we determined the level of 8-OHdG on the rat liver through immunohistochemical analysis. As shown in Figure 6c, numerous 8-OHdG-positive foci were found in the livers of AAF-treatment-only rats, whereas no 8-OHdG-positive foci were found in either the control group or the AAF plus NLE supplementation group. 

## 4. Discussion

Previous studies shown that NLE was beneficial for improving lipid metabolisms and alleviating liver damage in a high-fat diet treatment [11]. In addition, NLE prevented alcoholic steatohepatitis by reducing lipid synthesis, enhancing fatty acid transport responses, inhibiting hepatic lipid peroxidation, and facilitating anti-inflammation [12]. It has been proposed that chronic inflammation is the primary initiating factor responsible for many diseases including cancer. In the process of inflammation, mast cells and leucocytes are first recruited to produce ROS and various mediators such as cytokines, which further generate inflammatory cells to produce ROS. Overproduction of ROS imbalances the equilibrium of prooxidant/antioxidant, then causes oxidative stress which alters and damages many intracellular molecules such as protein, lipid, and nucleic acid. HCC may be starting from liver chronic inflammation, fibrosis, and cirrhosis to outcome the cancer. Chemical carcinogenesis is suggested to be a multistage process. AAF, an aromatic amine, exhibits its carcinogenic effect through the formation of DNA adducts, overproduction ROS, and induction of inflammatory factors. Our investigation found that NLE reduced AAF-induced oxidative stress, inflammation, fibrosis, and HCC. It implied that the components of NLE exerted chemopreventive role in multistage process which includes initiation, promotion, and progression. 

The highest incidence of HCC is found in Asia and sub-Saharan Africa. Most cases of HCC develop in a background of chronic liver damage such as hepatitis or fibrosis. Chronic liver damage causes a change in the hepatic microenvironment, thereby increasing the concentration of ROS and cytokines, which may be involved in the multistep process of hepatocarcinogenesis [34]. In the present study, we found that AAF treatment for 3 months induced hepatic fibrosis in rats. After being subjected to AAF treatment for 6 months, rats exhibited lipid metabolic disorder and hepatic damage and inflammation. In addition, AAF treatment for 6 months transformed hepatocytes and induced early hepatocarcinogenesis. Although NLE supplementation could not restore AAF-induced body weight loss and some of serum parameters such as γ-GT, AFP and TNF-α, but NLE reduced hepatic injury, inflammation, and hepatocarcinogenesis. These results suggest that NLE is effective in protecting the liver from AAF-induced hepatic injury and preventing further progression of carcinogenesis. 

Polyphenolic compounds such as flavonoids and phenolic acids are present in many vegetables, fruits, and herbs, certain of which have been reported to decrease the incidence and mortality of cancer [35]. Recently, ethanolic extract of lotus leaves containing catechin glycoside andisoquercitrin demonstrates antioxidative and anti-inflammatory effect, [36,37,38]. It has been reported that treated with 300 and 500 mg/kg of ethanolic extract of lotus leaves daily for 5 days protects CCl_4_-induced acute hepatotoxicity by evaluating serum parameters such as ALT, AST, and γGT, and hepatic antioxidant activity such as SOD and catalase [38]. Our study found that aqueous extract of lotus leaves (NLE) contained polyphenolic compounds such as gallic acid, catechin, isoquercitrin, and miquelianin. 2% NLE supplementation in diet was equal to about 160 mg/kg/day for 6 months, which was capable to reduce AAF-induced hepatotoxicity and hepatocarcinogenesis. Quercetin, a metabolite aglycone of isoquercitrin and miquelianin, has broad biophamcological effects such as hepatoprotection. Administration of 10–20 mg/kg quercetin daily for fourteen days reduces ethanol-induced liver injury in rats. The parameters such as ALT, AST, γGT, MDA, IL-6, and TNF-α were significantly reduced [39]. In addition, gallic acid (50 mg/kg) for five weeks reduces N-ntrosodiethylamine- induced hepatocellular carcinoma owing to regulate signal transducer through antioxidant and anti-inflammatory effects [40]. Basis on these reports and our results, we suggest that the polyphenol-rich components of NLE potentially provide benefits for blocking AAF-induced hepatocarcinogenesis through enhancing antioxidative potential and alleviating inflammation.

**Figure 7:**
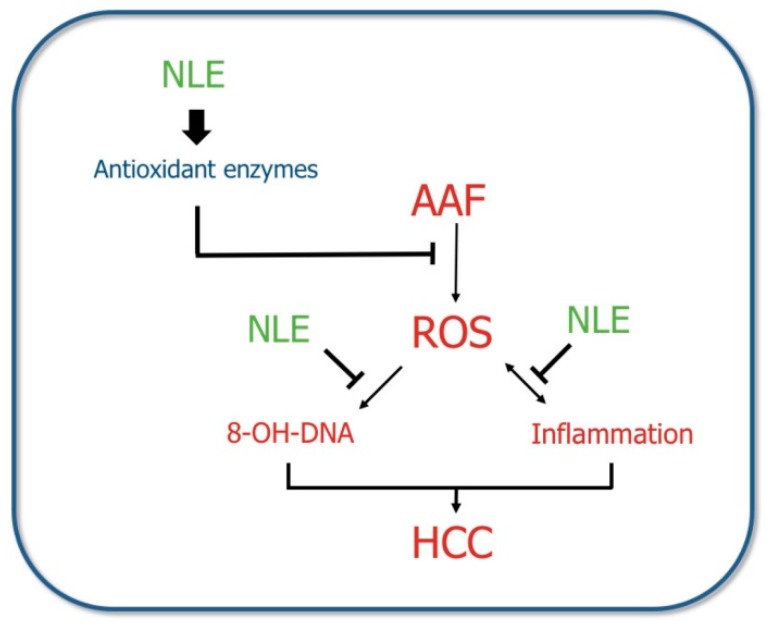
Mechanism of inhibition of hepatic damage by NLE.

The liver is a multifunctional organ that is responsible for detoxification and metabolic homeostasis. Thereafter, ROS are produced in liver cells as by-products of normal metabolic functions and detoxification. ROS may attack hepatocytes and lead to cell damage and inflammation. It has been suggested that ROS not only causes damage of the DNA but also influences epigenetic modification of gene expression, which may disturbances in the cell proliferation, differentiation, apoptosis, and angiogenesis [41]. Nrf2 is a transcription factor that regulates antioxidant defense gene expression in the liver. It is activated in response to oxidative stress and plays a protective role through target gene induction. In the present study, NLE supplementation increased the expression level of Nrf2 and its target genes such as catalase, SOD, and GPx, which contributed to the alleviation of liver injury and hepatocarcinogenesis. In addition, NLE contains large amounts of natural polyphenols, the cancer preventive properties of which may be attributed to their capacity for quenching ROS. Our results demonstrated that NLE prevents AAF-induced hepatocarcinogenesis by increasing the expression and activity of the antioxidant enzymes and blocking ROS formation and inflammation (Figure 7). Our findings are based on rat animal model, it still need further investigations to determine whether this also effective in human. 

## 5. Conclusions

NLE supplementation significantly alleviated AAF-induced hepatic injury and early hepatocarcinogenesis, which may result from decreasing AAF-induced inflammatory mediators and enhancing antioxidant enzyme expression. These results suggest that NLE could potentially be developed as a natural agent for preventing hepatocarcinogenesis. Whether NLE can perform a chemopreventive role in other cancer is worth study. In addition, since poor chemosensitivity is a major barrier to successful chemotherapy in HCC, the combination of NLE with chemotherapy for HCC treatment will be investigated in future.

## References

[1] Altekruse S.F., McGlynn K.A., Kickie L.A., Kleiner D.E. (2012). Hepatocellular carcinoma confirmation, treatment, and survival insurveillance, epidemiology, and end results registries, 1992–2008. Hepatology.

[2] Yu L.X., Ling Y., Wang H.Y. (2018). Role of nonresolving inflammation in hepatocellular carcinoma development and progression. NPJ Precis. Oncol..

[3] Fattovich G., Stroffolini T., Zagni I., Donato F. (2004). Hepatocellular carcinoma in cirrhosis: Incidence and risk factors. Gastroenterology.

[4] Zhang D.Y., Friedman S.L. (2012). Fibrosis-dependent mechanisms of hepatocarcinogenesis. Hepatology.

[5] Jung H.A., Kim J.E., Chung H.Y., Choi J.S. (2003). Antioxidant principles of *Nelumbo nucifera* stamens. Arch. Pharm. Res..

[6] Ling Z.Q., Xie B.J., Yang E.L. (2005). Isolation, characterization, and determination of antioxidative activity of oligomeric procyanidins from the seedpod of *Nelumbo nucifera* Gaertn. J. Agric. Food Chem..

[7] Mukherjee P.K., Das J., Saha K., Giri S., Pal M., Saha B. (1996). Antipyretic activity of *Nelumbo nucifera* rhizome extract. Indian J. Exp. Biol..

[8] Mukherjee P.K., Saha K., Pal M., Saha B. (1997). Effect of *Nelumbo nucifera* rhizome extract on blood sugar level in rats. J. Ethnopharmacol..

[9] Ono Y., Hattori E., Fukaya Y., Imai S., Ohizumi Y. (2006). Anti-obesity effect of *Nelumbo nucifera* leaves extract in mice and rats. J. Ethnopharmacol..

[10] Ho H.H., Hsu L.S., Chan K.C., Chen H.M., Wu C.H., Wang C.J. (2010). Extract from the leaf of nucifera reduced the development of atherosclerosis via inhibition of vascular smooth muscle cell proliferation and migration. Food Chem. Toxicol..

[11] Wu C.H., Yang M.Y., Chan K.C., Chung P.J., Ou T.T., Wang C.J. (2010). Improvement in high-fat diet-induced obesity and body fat accumulation by a *Nelumbo nucifera* leaf flavonoid-rich extract in mice. J. Agric. Food Chem..

[12] Tang C.C., Lin W.L., Lee Y.J., Tang Y.C., Wang C.J. (2014). Polyphenol-rich extract of *Nelumbo nucifera* leaves inhibits alcohol-induced steatohepatitis via reducing hepatic lipid accumulation and anti-inflammation in C57BL/6J mice. Food Funct..

[13] Chang C.H., Ou T.T., Yang M.Y., Huang C.C., Wang C.J. (2016). *Nelumbo nucifera* Gaertn leaves extract inhibits the angiogenesis and metastasis of breast cancer cells by downregulation connective tissue growth factor (CTGF) mediated PI3K/AKT/ERK signaling. J. Ethnopharmacol..

[14] Yang M.Y., Chang Y.C., Chan K.C., Lee Y.J., Wang C.J. (2011). Flavonoid-enriched extracts from *Nelumbo nucifera* leaves inhibits proliferation of breast cancer in vitro and in vivo. Eur. J. Integr. Med..

[15] Lin M.C., Kao S.H., Chung P.J., Chan K.C., Yang M.Y., Wang C.J. (2009). Improvement for high fat diet-induced hepatic injuries and oxidative stress by flavonoid-enriched extract from *Nelumbo nucifera* leaf. J. Agric. Food Chem..

[16] Lima K.G., Krause G.C., Schuster A.D., Catarina A.V., Basso B.S., De Mesquita F.C., Pedrazza L., Marczak E.S., Martha B.A., Nunes F.B. (2016). Gallic acid reduces cell growth by induction of apoptosis and reduction of IL-8 in HepG2 cells. Biomed. Pharmacother..

[17] Jagan S., Ramakrishnan G., Anandakumar P., Kamaraj S., Devaki T. (2008). Antiproliferative potential of gallic acid against diethylnitrosamine-induced rat hepatocellular carcinoma. Mol. Cell. Biochem..

[18] Shimizu M., Shirakami Y., Sakai H., Kubota M., Kochi T., Ideta T., Miyazaki T., Moriwaki H. (2015). Chemopreventive potential of green tea catechins in hepatocellular carcinoma. Int. J. Mol. Sci..

[19] Choi S., Lim T.G., Hwang M.K., Kim Y.A., Kim J., Kang N.J., Jang T.S., Park J.S., Yeom M.H., Lee K.W. (2013). Rutin inhibits B[a]PDE-induced cyclooxygensase-2 expression by targeting EGFR kinase activity. Biochem. Pharmacol..

[20] Chandra Y.P., Viswanathswamy A. (2018). Chemopreventive effect of Rutin against N-nitrosodiethylamine-induced and phenobarbital-promoted hepatocellular carcinoma in Wistar rats. Indian J. Pharm. Educ. Res..

[21] Huang G., Tang B., Tang K., Dong X., Deng J., Liao L., Liao Z., Yang H., He S. (2014). Isoquercitrin inhibits the progression of liver cancer in vivo and in vitro via the MAPK signalling pathway. Oncol. Rep..

[22] Wu C.H., Yang M.Y., Wang C.J. (2019). Quercetin-3-O-glucuronide inhibits doxorubicin resistance by reducing endoplasmic reticulum stress in hepatocellular carcinoma cells. J. Funct. Foods.

[23] Li W., Hao J., Zhang L., Cheng Z., Deng X., Shu G. (2017). Astragalin reduces hexokinase 2 through increasing miR-125b to inhibit the proliferation of hepatocellular carcinoma cells in vitro and in vivo. J. Agric. Food Chem..

[24] Strom S.C., Jirtle R.L., Michalopoulos G. (1983). Genotoxic effects of 2-acetylaminofluorene on rat and human hepatocytes. Environ. Health Perspect..

[25] Klöhn P.C., Massalha H., Neumann H.G. (1995). A metabolite of carcinogenic 2-acetylaminofluorene, 2-nitrosofluorene, induces redox cycling in mitochondria. Biochim. Biophys. Acta Bioenergy.

[26] Hsu J.D., Kao S.H., Chang-Che Tu C.C., Li Y.J., Wang C.J. (2009). Solanum nigrum L. Extract Inhibits 2-Acetylaminofluorene-Induced Hepatocarcinogenesis through Overexpression of Glutathione S-Transferase and Antioxidant Enzymes. J. Agric. Food Chem..

[27] Bergmeyer H., Herder M., Rej R. (1986). International Federation of Clinical Chemistry (IFCC). J. Clin. Chem. Clin. Biochem..

[28] Ohkawa H., Ohishi N., Yagi K. (1979). Assay for lipid peroxides in animal tissues by thiobarbituric acid reaction. Anal. Biochem..

[29] Lee H.J., Chen C.C., Chou F.P., Wu C.H., Lai F.S., Yang M.Y., Wang C.J. (2010). Water extracts from *Nelumbo nucifera* leaf reduced plasma lipids and atherosclerosis in cholesterol-fed rabbits. J. Food Biochem..

[30] Lawrence R.A., Burk R.F. (1976). Glutathione peroxidase activity in selenium-deficient rat liver. Biochem. Biophys. Res. Commun..

[31] Marklund S., Marklund G. (1974). Involvement of the superoxide anion radical in the autoxidation of pyrogallol and a convenient assay for superoxide dismutase. Eur. J. Biochem..

[32] Aebi H. (1984). Catalase in vitro. Methods Enzymol..

[33] Li T., Zhao X.P., Wang L.Y., Gao S., Zhao J., Fan Y.C., Wang K. (2013). Glutathione S-transferase P1 correlated with oxidative stress in hepatocellular carcinoma. Int. J. Med. Sci..

[34] Tu T., Budzinska M., Maczurek A., Cheng R., Di Bartolomeo A., Warner F., McCaughan G., McLennan S., Shackel N. (2014). Novel aspects of the liver microenvironment in hepatocellular carcinoma pathogenesis and development. Int. J. Mol. Sci..

[35] Carocho M., Ferreira I.C.F.R. (2013). The role of phenolic compounds in the fight against cancer—A review. Anticancer Agents Med. Chem..

[36] Xu H.C., Wang M.Y. (2014). Effect of flavonoids from Lotus (*Nelumbo nuficera* Gaertn) leaf on biochemical parameters related to oxidative stress induced by exhaustive swimming exercise of mice. Biomed. Res..

[37] Park E., Kim G.D., Go M.S., Kwon D., Jung I.K., Auh J.H., Kim J.H. (2017). Anti-inflammatory effects of *Nelumbo* leaf extracts and identification of their metabolites. Nutr. Res. Pract..

[38] Huang B., Ban X., He J., Tong J., Tian J., Wang Y. (2010). Hepatoprotective and antioxidant activity of ethanolic extracts of edible lotus (*Nelumbo nucifera* Gaertn.) leaves. Food Chem..

[39] Chen X. (2010). Protective effects of quercetin on liver injury induced by ethanol. Pharmacogn. Mag..

[40] Aglan A.H., Ahmed H.H., EI-Toumy S.A., Mahmoud N.S. (2017). Gallic acid against hepatocellular carcinoma: An integrated scheme of the potential mechanisms of action from in vivo study. Tumor Biol..

[41] O’Rourke J.M., Sagar V.M., Shah T., Shetty S. (2018). Carcinogenesis on the background of liver fibrosis: Implications for the management of hepatocellular cancer. World J. Gastroenterol..

